# Enhancing instantaneous oxygen uptake estimation by non-linear model using cardio-pulmonary physiological and motion signals

**DOI:** 10.3389/fphys.2022.897412

**Published:** 2022-08-25

**Authors:** Zhao Wang, Qiang Zhang, Ke Lan, Zhicheng Yang, Xiaolin Gao, Anshuo Wu, Yi Xin, Zhengbo Zhang

**Affiliations:** ^1^ Medical School of Chinese PLA, Beijing, China; ^2^ School of Life Science, Beijing Institute of Technology, Beijing, China; ^3^ Beijing SensEcho Science and Technology Co Ltd, Beijing, China; ^4^ PAII Inc., Palo Alto, Santa Clara, CA, United States; ^5^ Institute of Sports Science, General Administration of Sport of China, Beijing, China; ^6^ The Paul G. Allen School of Computer Science and Engineering, University of Washington, Seattle, WA, United States; ^7^ Center for Artificial Intelligence in Medicine, Chinese PLA General Hospital, Beijing, China

**Keywords:** oxygen uptake, machine learning, wearable sensor, XGBoost, heart rate, respiration

## Abstract

Oxygen uptake (VO_2_) is an important parameter in sports medicine, health assessment and clinical treatment. At present, more and more wearable devices are used in daily life, clinical treatment and health care. The parameters obtained by wearables have great research potential and application prospect. In this paper, an instantaneous VO_2_ estimation model based on XGBoost was proposed and verified by using data obtained from a medical-grade wearable device (Beijing SensEcho) at different posture and activity levels. Furthermore, physiological characteristics extracted from single-lead electrocardiogram, thoracic and abdominal respiration signal and tri-axial acceleration signal were studied to optimize the model. There were 29 healthy volunteers recruited for the study to collect data while stationary (lying, sitting, standing), walking, Bruce treadmill test and recuperating with SensEcho and the gas analyzer (Metalyzer 3B). The results show that the VO_2_ values estimated by the proposed model are in good agreement with the true values measured by the gas analyzer (R^2^ = 0.94 ± 0.03, *n* = 72,235), and the mean absolute error (MAE) is 1.83 ± 0.59 ml/kg/min. Compared with the estimation method using a separate heart rate as input, our method reduced MAE by 54.70%. At the same time, other factors affecting the performance of the model were studied, including the influence of different input signals, gender and movement intensity, which provided more enlightenment for the estimation of VO_2_. The results show that the proposed model based on cardio-pulmonary physiological signals as inputs can effectively improve the accuracy of instantaneous VO_2_ estimation in various scenarios of activities and was robust between different motion modes and state. The VO_2_ estimation method proposed in this paper has the potential to be used in daily life covering the scenario of stationary, walking and maximal exercise.

## Introduction

Assessment of the functional capacity of the cardiovascular system is essential in sports medicine and clinical settings (Kaminsky et al., 2019). Oxygen uptake (VO_2_), which indicates an individual’s aerobic capacity (Hill and Lupton, 1923), provides important information for monitoring exercise intensities and changes in an athlete’s fitness during training. At the same time, as Metabolic Equivalency Task (MET) (Negus et al., 1987), VO_2_ is a standard indicator of individual metabolic rate and subsequent physical activity. It is used to provide general medical thresholds and guidelines for people with chronic diseases such as obesity and Type 2 diabetes (Hupin et al., 2015). In addition, steady state VO_2_ measurements are considered to be the gold standard for estimating energy expenditure (EE) in light to moderate steady motion (Scott, 2005; Altini et al., 2015). The peak VO_2_ reached during incremental motion is called the maximum VO_2_ (as VO_2_max). In physical training, VO_2_max and its derivatives [including vVO_2_max (Billat, 2001) and TLim-vVO_2_max (Fernandes et al., 2006)] are widely used in physical training programs and have been shown to be helpful in improving athletes’ performance.

The traditional VO_2_ measurement methods mainly focus on direct calorimetry in metabolic chambers (Kenny et al., 2017), double-label water (Hills et al., 2014) or indirect calorimetry (Leonard, 2012) with face masks as the “gold standard”, which are not suitable for daily exercise due to the need for expensive gas analysis, ventilation equipment and medical care. Some researchers have refined existing devices such as COSMED K5 (Guidetti et al., 2018), VO_2_ Master (Montoye et al., 2020) and Jaeger (Díaz et al., 2008) to develop portable calorimetric systems capable of accurately measuring VO_2_ in outdoor conditions. However, the high cost and highly visible components such as masks and gas analyzers limit the use of portable calorimetric systems in non-laboratory settings.

Heart rate (HR) was a low-cost and non-invasive method of estimating VO_2_ because of its strong linear relationship with VO_2_ during a large amount of aerobic exercise (Livingstone, 1997). Therefore, many studies have proposed their models for predicting VO_2_ and VO_2_max using HR (Pulkkinen et al., 2004; Nevill and Cooke, 2016; Mazzoleni et al., 2018; Lanferdini et al., 2020). However, the model performance of predicting VO_2_ only with HR is limited due to the ambiguous relationship between HR and VO_2_ at rest and low intensity motion, as well as transitions between different activities (Pulkkinen et al., 2004). In addition, HR measurements are susceptible to both internal [stress, emotions, etc. (Lanferdini et al., 2020)] and external [Wrist-based Photoplethysmography assessment of HR is affected by the environment, skin, sweat, etc. (Spurr et al., 1988)] factors. The Flex-HR model is one of the most commonly used HR-based methods for VO_2_ estimation in the field. Considering the non-linear relationship of HR-VO_2_ during low intensity motion, bilinear model was used to improve accuracy (Spurr et al., 1988). Acceleration (ACC) sensors can detect postural motion information to identify the type and intensity of motion (Crouter et al., 2010; Ellis et al., 2014), which, in combination with HR, improves the accuracy of the VO_2_ estimation (Strath et al., 2005). Andrew et al. (Cook et al., 2018) estimated real-time VO_2_ using ACC, HR and demographic characteristics as inputs to a multiple linear regression model. A total of 42 subjects (including healthy, athletic and obese) were recruited in the experiment for the Bruce treadmill experiment, which showed a strong linear correlation between the predicted VO_2_ and the actual VO_2_ (r = 0.93). Respiratory signals can represent changes in lung ventilation during exercise (Gastinger et al., 2014), and a linear relationship between the pulmonary ventilation and VO_2_ has been found to be superior to that of HR (Gastinger et al., 2010). Andrea et al. (Nicolò et al., 2017) suggested that researchers need to focus on the potential of respiratory signal in exercise training to identify EE in subjects’ daily activities through the combination of HR and respiration rate (RR), which is more accurate than using a HR model alone. Recognizing that respiration signals are another key factor in the high correlation with VO_2_. Beltrame et al. (2017) considered not only HR and ACC information, but also RR and the calculated per minute ventilation (VE) based on the respiratory signals collected by the wearable shirt. However, Beltrame only considered daily routines and low-intensity exercise, and the subjects in the study did not reach the level of VO_2_max.

With the development of wearable devices, it has become a promising method to predict VO_2_ through physiological parameters obtained by devices such as smart watches or shirts. For example, sports watches like Apple watch (Falter et al., 2019) and Fitbit (Sasaki et al., 2015) can track EE in real time, which is non-intrusive and portable. A representative wearable smart shirt Hexoskin (Beltrame et al., 2017) can obtain a wide range of physiological parameters of the wearer to improve the accuracy of VO_2_ estimate mentioned in the previous paragraph. In addition, many researchers have used self-designed portable devices (Lu et al., 2019) to collect physiological signals for VO_2_ and VO_2_max prediction. Shandhi et al. (2020) developed a novel wearable patch that can obtain seismocardiogram (SCG), electrocardiogram (ECG) and atmospheric pressure (AP) signals, and they extracted features from these signals to estimate the VO_2_ with the R^2^ of 0.77.

So far, there have been some researches on the real-time prediction of VO_2_ based on easily available physiological signals. However, there are still some problems to be solved. First, VO_2_ still cannot be accurately estimated in rest, low-intensity exercise, and maximal exercise. Second, although some consumer-grade watches provide the function of giving VO_2_ in daily activities, they are probably not accurate enough for sports or health care (Murakami et al., 2019; Passler et al., 2019). In order to further solve the above issues and improve the accuracy of VO_2_ estimation, the specific work of this study is summarized as follows:

1) Using wearable devices to simultaneously record ECG, respiration and ACC monitoring data, and extract features, including pulmonary ventilation related parameters, to establish a machine learning model to predict dynamic VO_2_ regardless of the current activity type.
2) The introduction of respiration features improves the effect of the VO_2_ estimation model in rest and low-intensity exercise. The features extracted from ACC signals reflect the exercise intensity of the subjects and play an important role in the instantaneous VO_2_ estimation.
3) The training data source experiment includes a variety of rest states, continuous different exercise mode stages including low-intensity, high-intensity exercise stages and exercise recovery stages, almost covering the activity mode in daily activities.
4) The effects of input parameters, gender, exercise intensity, and individual differences on model performance were comprehensively discussed in the study, providing more insight into the accurate estimation of VO_2_ in daily life and exercise.

## Materials and methods

### Participants and data acquisition

A total of 31 healthy young volunteers were recruited for this study, mainly from non-sports postgraduate students in colleges and universities, including 19 males and 12 females. Each participant in the study followed the protocol approved by the IRB review board (IRB number: S2018-095-01) and approved the written informed consent procedure. Demographic information, including age, sex, weight, and height, was collected through a questionnaire. Due to the high exercise intensity during the experiment, some subjects experienced equipment dropping or ECG leads dislocation. Therefore, the final number of subjects with complete data collected for the entire procedure was 29 (17 male, 12 female). The demographic information is summarized in Table 1.

**TABLE 1:** The demographic information of the subjects [mean (sd)].

	All (29)	Male (17)	Female (12)
Age (Years)	24.19 (2.82)	24.47 (2.70)	23.25 (1.83)
Height (cm)	169.97 (7.64)	174.53 (3.91)	162.83 (6.22)
Body mass/weight (kg)	63.34 (10.31)	70.19 (6.93)	53.53 (5.46)
BMI (kg/m^2^)	21.74 (2.16)	22.94 (1.95)	20.17 (1.21)

**FIGURE 1:**
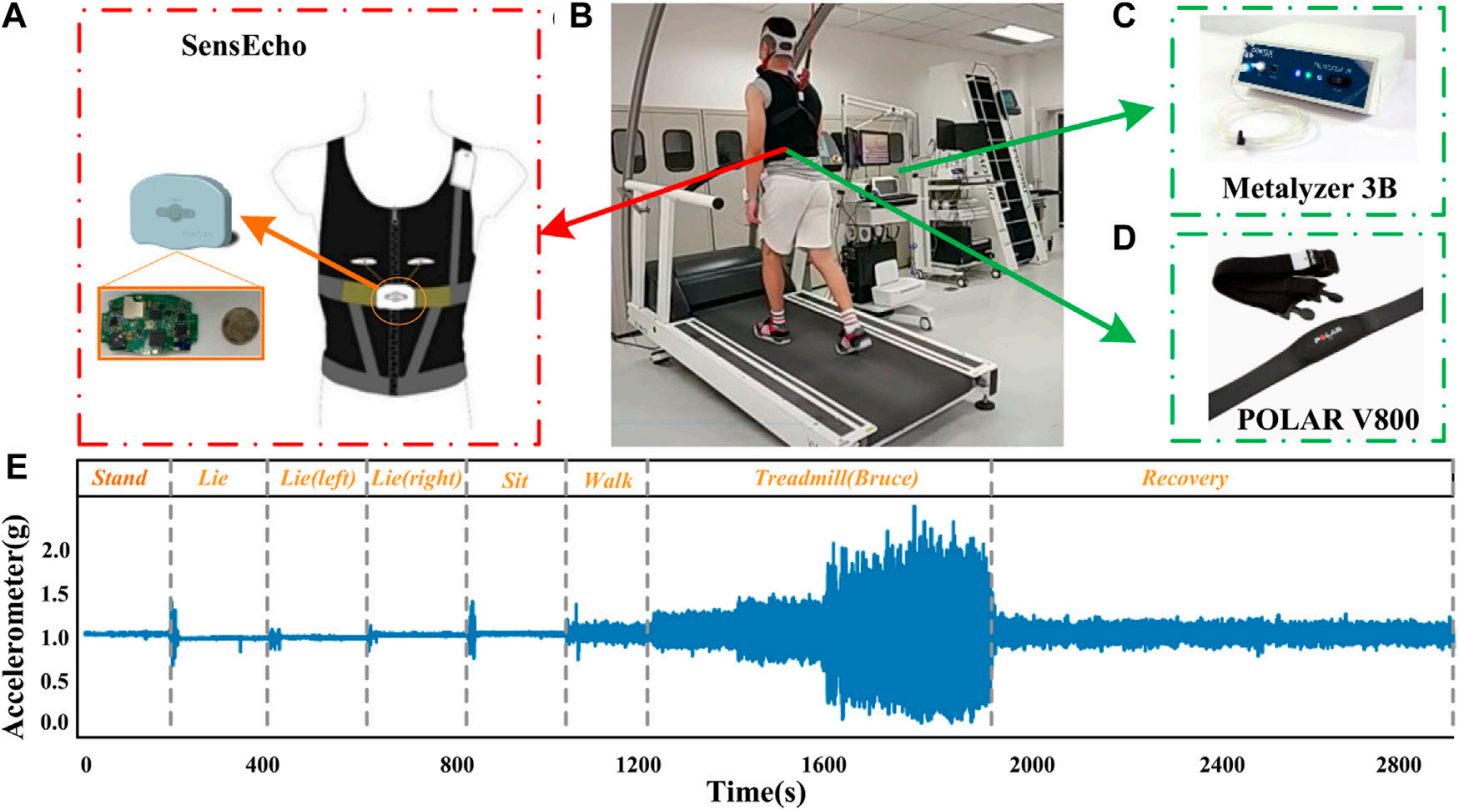
**(A)** SensEcho wearable device. **(B)** A subject configured with both the wearable vest and the gas analyzer (Metalyzer 3B). **(C)** Metalyzer 3B. **(D)** POLAR V800. **(E)** Representative chest acceleration response during the experiments.

During the 2 hours before the experiments, participants were prohibited from drinking, eating, or performing excessive physical activity. Upon arrival at the test site, the subjects filled out a registration form and a cardiovascular risk questionnaire under the guidance of the researchers. The doctor assessed the potential exercise risk based on the results of the questionnaire, and then the subjects wore the SensEcho and Metalyzer 3B monitoring system under the supervision of a researcher, as shown in Figure 1B. The experiment includes three phases: rest, activity and recovery. During the resting period, subjects were in several postures, including standing, lying with straight legs, lying on the left side, lying on the right side, and sitting upright. In each posture, each subject performed normal breathing (1 min), deep breathing (1 min), talking (30 s), and fast breathing (30 s), with 30 s of rest and adjustment between postures. The duration of the entire phase was 17 min.

After completing the resting phase, each subject walked on the treadmill to warm up for 3 min, and then followed the Bruce exercise protocol, which is widely used in treadmill-based exercise tests (Hamlin et al., 2012) and clinical examinations (Bruce et al., 1973). This is a progressive test to reach the participant’s maximum tolerable activity level. Each phase lasts for 3 min, as shown in Table 2. During the Bruce test, if the subject is exhausted, the researcher will stop the treadmill, and the subject will enter the recovery phase and walk slowly on the treadmill until VO_2_ returns to his/her warm-up level. The duration of this phase does not exceed 20 min. Figure 1E shows the representative acceleration of the chest response throughout the experiment.

**TABLE 2:** The Bruce exercise protocol.

Level	Time (min)	Speed (km/h)	Incline (%)
1	1–3	2.74	10
2	4–6	4.02	12
3	7–9	5.47	14
4	10–12	6.76	16
5	13–15	8.05	18
6	16–18	8.85	20
7	19–21	9.65	22

Remarks: Exhaustion criteria: a) The VO2 reaches its peak; b) The respiratory quotient ≥1.10 for adults and ≥1.00 for children; c) HR ≥ 180 BPM; d) The subject was unable to continue exercise tests.

### Hardware

The Metalyzer 3B (Cortex, Germany) is a commonly used cardiopulmonary function testing device (Meyer et al., 2001). It uses a mixed gas or heart-to-heart testing method to collect vital signs parameters such as RR, HR, respiratory exchange rate in real time. It is widely used in the comparison of cardiopulmonary function experiments under different populations and conditions (Shieh et al., 2010; Xiong et al., 2013). The device consists of two parts: lung function detection and heart rate monitoring. We follow the “Two-Point Gas Calibration” method on Page 41 in the Operator’s Manual MetaLyzer 3B (CORTEX Biophysik, 2021). The span gas with 15% O_2_, 5% CO_2_, bal. in N_2_ was used to calibration the gas analyzer. The Metalyzer 3B (Cortex, Germany) shown in Figure 1C was used to collect VO_2_ data in seconds (*fs = 1* *Hz*), and the POLAR V800 shown in Figure 1D was used as the gold standard to collect HR data (*fs = 1* *Hz*). The subject is required to wear a matching face mask during the measurement.

The SensEcho (SensEcho, Beijing SensEcho Technology Co., Ltd.) we used in the experiment is a medical-grade wearable vest embedded with multiple biosensors to monitor various vital signs (Xu et al., 2020; Wang et al., 2021; Wang et al., 2022). The SensEcho system consists of three parts, namely, the sensors that collect physiological parameters, the wireless data transmission network and the central monitoring system. The ECG signals are collected through three electrode patches. Two induction belts are embedded in the chest and abdomen of the vest to collect the chest and abdomen motion signals to give an estimate of respiratory rate (RR). The errors of HR and RR measurement are both within ±2BPM. SensEcho uses ultra-low-power tri-axial accelerometer MMA7260 (Freescale Inc., TX, United States) to collect posture and motion information with an accuracy of 8 mg/LSB (Least Significant Bit). The main control chip of the system is an ultra-low-power ARM cortex-m3 MCU (EFM32GG330, Silicon Labs, United States) with a power consumption of 100 mW. Figure 1A shows SensEcho wearable vest. The system also provides local and cloud data storage solutions. When the cloud storage is unstable or unavailable, the local storage can be activated to save the original data in a 2 GB integrated flash drive. The single-lead ECG (sampling frequency *f*_*s*_ = 200 Hz), respiratory signal (*f*_*s*_ = 25 Hz), and tri-axial accelerometer data (*f*_*s*_ = 25 Hz) were collected by medical-grade wearable devices, as shown in Figure 1A.

### Data pre-processing and features extraction

**FIGURE 2:**
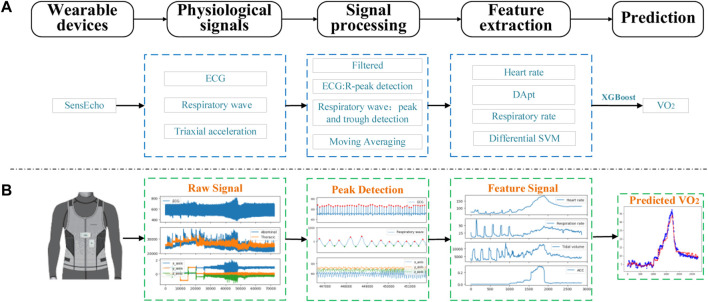
The process of signal acquisition, preprocessing and estimation with SensEcho wearable device. **(A)** A flow chart of the entire experiment. **(B)** An example of the visualization of key signal processes. (Abbreviations: ECG, Electrocardiograph; DA_pt_, the difference between the amplitude of the wave peaks and the amplitude of the troughs, SVM, the signal vector magnitude of triaxial accelerometer).

In the data preprocessing stage, this article performs filtering and noise reduction operations on each signal from SensEcho, and then extracts heart rate characteristics from SensEcho’s ECG, respiration rate and lung ventilation related characteristics from respiration signals, and exercise intensity from ACC data Features, as shown in Figure 2.

The original ECG signal from the wearable vest was filtered by a finite impulse response (FIR) bandpass filter with cutoff frequencies of 3–45 Hz, which were selected for the ECG signal to reduce ST-band interference and to amplify the R-wave for better R-peak detection in the subsequent signal processing steps. The R-peaks detection used the classical E. P. Limited algorithm (Hamilton, 2002), the r-r interval was calculated by the difference of adjacent R peaks, and the HR with a sampling rate of 1 Hz was calculated with a time window of 4s and a moving step of 1s. The age-based maximum HR (HR_max_) (Tanaka et al., 2001)and the ratio of current HR to age-based maximum HR (HR%) were got according to the following Eqs 1–3.

HR=60/(r−r interval) (1)

HRmax=208−0.7×age (2)

$$\text{HR\%}=\frac{\text{HR}}{{\text{HR}}_{\text{max}}}\text{∗}100\text{\%}$$ (3)

The wavelet decomposition technologies were applied to remove the offset effects in the breathing signals. Butterworth band-pass filter with the frequency of 0.1–0.35 Hz was applied to remove the high frequency noises. The NeuroKit (Makowski et al., 2020) program package was used to detect the respiratory wave peaks. The R-R interval was calculated by using the difference of adjacent respiratory wave peak. The difference amplitude ( ${\text{DA}}_{\text{pt}}$ ) of each breath was calculated from the difference between the amplitude of the wave peaks ( ${\text{AM}}_{\text{rr}\_\text{peaks}}$ ) and the amplitude of the troughs ( ${\text{AM}}_{\text{rr}\_\text{trough}}$ ).

RR=60/(R−R interval) (4)

$${\text{DA}}_{\text{pt}}={\text{AM}}_{\text{rr}\_\text{peaks}}-{\text{AM}}_{\text{rr}\_\text{trough}}$$ (5)

The low-pass filter with 0.32 Hz was used to reduce the motion artifacts of ACC. After the filtering, the signal vector magnitude (SVM) was calculated, using the processed triaxial acceleration that had been obtained, with the formula as follows:

$$\text{SVM}\left(\text{i}\right)=\sqrt{\text{x}\_\text{acc}{\left(\text{i}\right)}^{2}+\text{y}\_\text{acc}{\left(\text{i}\right)}^{2}+\text{z}\_\text{acc}{\left(\text{i}\right)}^{2}}$$ (6)

In the above formula, the output of the ACC refers to x_acc, y_acc, and z_acc, respectively. The mean absolute value of differential SVM (MADs) is calculated by the following formula, which represents the intensity of exercise.

$$\text{MADs}\left(\text{i}\right)=\frac{1}{\text{T∗}25-1}\times \sum_{\text{i}=1}^{\text{T∗}25-1}\left[\text{SVM}\left(\text{i}+1\right)-\text{SVM}\left(\text{i}\right)\right]$$ (7)

In the formula, T is the time length (s) for MADs calculation, which is 1s in this paper.

Since the sampling rate of the gas analyzer is 1 Hz, to synchronize with it, we adopted monotone cubic interpolation to form the RR, $DA_{pt}$ and MADs with sampling rate of 1 Hz. Finally, we smoothed the synchronized VO_2_, HR, RR, $DA_{pt}$ and MADs with 31 point moving average window to reduce interference noise. We used python 3.6 to conduct all data pre-processing and feature extraction steps.

### Regression model

XGBoost (Extreme Gradient Boosting) (Chen et al., 2015) is a machine learning technique for regression and classification problems. It is based on the Gradient Boosting Decision Tree (GBDT), an open-source machine learning project. In XGBoost regression model, the result of the prediction is the sum of the scores predicted by K trees, as shown in the formula below:

$${\hat{\text{y}}}_{\text{i}}=\sum_{\text{k}=1}^{\text{k}}{\text{f}}_{\text{k}}\left({\text{x}}_{\text{i}}\right),{\text{f}}_{\text{k}}\in \text{F}$$ (8)

In the above formula, ${\hat{\text{y}}}_{\text{i}}$ is the i-th predicted result, ${\text{x}}_{\text{i}}$ is the i-th training sample, ${\text{f}}_{\text{k}}\left({\text{x}}_{\text{i}}\right)$ is the score for the k-th tree, and F is the space of functions containing all regression trees.

The loss function is represented by ${\hat{\text{y}}}_{\text{i}}$ and the true value ( $y_{\text{i}}$ ). It is used to measure the suitability of the model to the training data set.

$$\text{L}=\sum_{i=1}^{n}\text{l}\left({\text{y}}_{\text{i}},{\hat{\text{y}}}_{\text{i}}\right)$$ (9)

The objective function to be optimized is given by the following formula:

$$\text{OBJ}=\sum_{i=1}^{n}\text{l}\left({\text{y}}_{\text{i}},{\hat{\text{y}}}_{\text{i}}\right)+\sum_{\text{k}=1}^{\text{K}}\text{Ω}\left({\text{f}}_{\text{k}}\right)$$ (10)

The $\sum_{\text{k}=1}^{\text{K}}\text{Ω}\left({\text{f}}_{\text{k}}\right)$ is an item that penalizes the complexity of the model and prevent overfitting. As the complexity of the model increases, a corresponding score is deducted.

Compared with GBDT, XGBoost has many algorithm and engineering improvements. XGBoost penalizes more complex models through LASSO (L1) and Ridge (L2) regularization to prevent overfitting (Morde, 2019). XGBoost naturally acknowledges the sparsity of the input by automatically learning to determine the maximum missing value based on training losses and to process the different types of sparse patterns in the data more efficiently (Chen et al., 2015). Therefore, it has been widely used in many machine learning competitions and achieved good results.

### Optimize hyperparameters

Firstly, the data set was divided into training set and validation set by the method of leaving one. Secondly, for each training set, the optimal parameters were selected by the method of grid search and five-fold cross-validation. Finally, the optimal parameter model was applied to the validation set to obtain the result. All steps were implemented in Python 3.6.

### Different feature set

For convenience, we named the features of subject demographic information (including age, gender, and BMI) as SDI, and added a new feature RD, which includes $RR\;and\;DA_{pt}$ . To explore the influence of different input parameters on model performance and further investigate the optimal parameter combination to predict VO_2_. In this work, we have designed multiple input combinations for different models: HR% + SDI, RD + SDI, MADs + SDI, HR% + RD + SDI, HR% + MADs + SDI, MADs + RD + SDI, HR% + RD + MADs + SDI.

### Data analysis

Leave-one-subject-out (LOSO) cross-validation was performed on *n* subjects. In each round, XGBoost regressor trained on the data from *n-1* subjects, and the remaining sample is used as the test set in which the VO_2_ of the left-out subject was predicted. The process was repeated *n-1* times with a different subject excluded each time. Performance of the different regression models and input parameters were evaluated using mean absolute error (MAE, ml/kg/min):

$$\text{MAE}=\;\frac{1}{N}\times \sum_{i=1}^{N}\left|VO_{2,true}\left(i\right)-VO_{2,esti}\left(i\right)\right|\;$$ (11)

In the above formula, N was the numbers of $VO_{2,esti}$ .

The coefficients of determination (R^2^) and Bland-Altman plot were used to analyze the consistency between the estimated VO_2_ ( $VO_{2,esti}$ ) and the true VO_2_ ( $VO_{2,true}$ ). All data analysis was carried out *via* Python (version 3.6).

### Statistics analysis

Firstly, to explore the influence of different hyperparameters on the VO_2_ prediction accuracy of different models with the same input characteristics, 1) the accuracy of LR, RF and XGB models in VO_2_ prediction was compared when the input characteristics were HR%+RD + MADs + SDI, 2) the accuracy of LR and XGB for VO_2_ prediction was compared when the input characteristics were HR% + SDI, RD + SDI, MADs + SDI, HR% + RD + SDI, HR% + MADs + SDI, MADs + RD + SDI. Secondly, to study the influence of different hyperparameters on the model, three hyperparameters were selected. On the premise that the other two hyperparameters were fixed, the influence of the change of the other hyperparameter on the prediction VO_2_ error was compared. Thirdly, to investigate the importance of different input features, we compared the VO_2_ prediction error with and without of the input features. Fourthly, to explore the influence of gender on the prediction accuracy of the model, the MAE of VO_2_ prediction was compared between the same gender as the training set and different gender as the training set. Finally, to compare the stability of the proposed model and the activity-specific model, the differences of VO_2_ prediction between the two models in rest (Stand, Lie, Lying on the left side, Lying on the right side, Sit), Walk, Run and recovery states were compared.

Independent sample t-test was used for comparison between two groups. One-way ANOVA was used for comparison between multiple groups. Additionally, for the post-hoc testing, we applied the Tukey HSD test for comparisons between groups. The *p*-value for one-way ANOVA is less than 0.05 indicate that at least one of the treatment groups differs from the others. In our study, we considered that a *p* < 0.05 was statistically significant.

## Results

### Comparison of different regression models and inputs

Linear regression (LR), random forest (RF), and XGBoost regression models were applied, and the XGBoost model worked best throughout the experiments with HR, RD, MADs, and SDI as model inputs. The MAE of VO_2_ predicted by these three models was significantly different (*p* < 0.05), but there was no significant difference of R^2^ (*p* > 0.05). Compared with LR and RF model, MAE of XGBoost model decreased by 0.74 ml/kg/min (*p* < 0.05) and 0.23 ml/kg/min (*p* > 0.05) respectively, and R^2^ increased by 0.06 (*p* > 0.05) and 0.02 (*p* > 0.05) respectively.

Further, the effects of different input signals on the accuracy of LR and XGBoost models were compared. Compared with LR, when the input signal was HR% + SDI, the MAE of XGBoost decreased by 0.20 ml/kg/min (*p* > 0.05), and R^2^ decreased by 0.01 (*p* > 0.05). When the input signal was RD + SDI or MADs + SDI, the MAE of XGBoost decreased by 1.03 ml/kg/min (*p* < 0.05) and 0.88 ml/kg/min (*p* > 0.05) respectively, and R^2^ increased by 0.11 (*p* < 0.05) and 0.08 (*p* < 0.05) respectively. When the input signal was HR%+RD + SDI, RD + MADs + SDI, or HR%+MADs + SDI, the MAE of XGBoost decreased (*p* < 0.05) by 0.82 ml/kg/min, 0.68 ml/kg/min, and 0.49 ml/kg/min, and R^2^ increased by 0.05 (*p* > 0.05), 0.06 (*p* < 0.05), and 0.05 (*p* < 0.05). The mean and standard deviation of MAE and R^2^ of different models and inputs were shown in Table 3.

**TABLE 3:** The MAE and R^2^ of different models and different input parameters [mean (sd)].

Models	Inputs	MAE (ml/kg/ml)	R^2^
LR	HR%+SDI	4.24 (1.45)	0.73 (0.17)
	RD + SDI	4.91 (0.94)	0.59 (0.19)
	MADs + SDI	3.58 (0.65)	0.75 (0.12)
	HR%+RD + SDI	3.94 (1.16)	0.77 (0.12)
	RD + MADs + SDI	2.90 (0.57)	0.83 (0.08)
	HR%+MADs + SDI	2.69 (0.81)	0.87 (0.08)
	HR%+RD + MADs + SDI	2.57(0.70)	0.88(0.06)
RF	HR%+SDI	4.20 (1.38)	0.68 (0.18)
	RD + SDI	4.33 (1.48)	0.58 (0.25)
	MADs + SDI	3.74 (0.78)	0.68 (0.13)
	HR%+RD + SDI	3.30 (1.03)	0.79 (0.11)
	RD + MADs + SDI	2.55 (1.14)	0.86 (0.11)
	HR%+MADs + SDI	2.52 (0.61)	0.88 (0.05)
	HR%+RD + MADs + SDI	2.06(0.43)	0.92(0.03)
XGBoost	HR%+SDI	4.04 (1.77)	0.72 (0.19)
	RD + SDI	3.88 (1.22)	0.70 (0.17)
	MADs + SDI	2.70 (0.58)	0.83 (0.06)
	HR%+RD + SDI	3.12 (1.21)	0.82 (0.11)
	RD + MADs + SDI	2.22 (0.76)	0.89 (0.07)
	HR%+MADs + SDI	2.20 (0.67)	0.92 (0.05)
	*HR%+RD + MADs + SDI*	1.83 (0.59)	0.94 (0.03)

The results show that in both LR and XGBoost models, the combination of multiple parameters reduces the MAE of estimated VO_2_ compared with using HR, RD, or MADs alone as inputs, while the XGBoost regression model performs better compared with the LR model either using a single feature or different combinations of multi-signal features as inputs. This is in line with expectations, as the linear relationship between individual metrics (e.g., HR, RR) and VO_2_ does not always hold under different states of motion, and the combination of these features is effective in reducing the VO_2_ prediction error compared with using HR%, RD, or MADs features alone as inputs.

### Effect of different parameters on the accuracy of XGBoost model

To explore the influence of parameters in the XGBoost model on the result estimation error, three hyper-parameters, learning rate (L_rate_), the number of trees (N_tree_), and max deep (D_max_) of the model were investigated in terms of measures of MAE. For this purpose, we change one of the hyper-parameters with all the others fixed. The effect of these important parameters of XGBoost on the accuracy of $VO_{2,esti}$ was shown in Table 4, and it was found that N_tree_ and L_rate_ had a greater effect on the results than the D_max_. The MAE of VO_2_ predicted by eight combinations of three parameters has significant difference (*p* < 0.05). Compared with 10 trees, when the D_max_ and L_rate_ of 50 trees were (1, 1), (1, 0.1), (5, 0.1), the MAE decreased by 18.50% (*p* < 0.05), 55.67% (*p* < 0.05), 57.93% (*p* < 0.05) respectively. Compared with D_max_ of 1, when the N_tree_ and L_rate_ of D_max_ of five were (10, 1) and (50, 0.1), the MAE decreased by 14.94% (*p* < 0.05) and 17.33% (*p* < 0.05) respectively. Compared with the L_rate_ of 1, the MAE decreased by 46.61% (*p* < 0.05), 47.87% (*p* < 0.05) and 24.90% (*p* < 0.05), when the N_tree_ and D_max_ of the L_rate_ of 0.1 were (10, 1), (10, 5), and (50, 5) respectively. Thus, the input parameters have a significant impact on the MAE in the results. It is important to adjust the parameters, and the optimal parameters will have a large improvement in the accuracy of the model.

**TABLE 4:** The results of different parameter for XGBoost model (mean (sd)).

(*N*_*tree*_ *, D*_*max*_ *, L*_*rate*_)	MAE
***10*,*1*,*1***	2.68 (0.34)
***10*,*5*,*1***	2.28 (0.66)
***50*,*1*,*1***	2.19 (0.47)
***50*,*5*,*1***	2.45 (0.78)
***10*,*1*,*0.1***	5.02 (1.35)
***50*,*1*,*0.1***	2.23 (0.62)
***10*,*5*,*0.1***	4.38 (1.21)
***50*,*5*,*0.1***	1.84 (0.52)

### Contributions of different input parameters under various activity types

In this paper, further discussions have been carried upon the results of the XGBoost model, as shown in Table 5, which shows the VO_2_ prediction results of various types of activities under different input parameters. Due to the poor linear correlation between VO_2_ and HR in the rest state, it is difficult to do the accurate prediction, so several rest scenarios were designed in the experiment. The results of each scenario were analyzed to explain how the input parameters affect the accuracy of VO_2_ in different states. The MAE of VO_2_ predicted by seven combination parameters (HR%+SDI, RD + SDI, MADs + SDI, HR%+RD + SDI, RD + MADs + SDI, HR%+MADs + SDI, HR% + RD + MADs + SDI) as inputs was significant difference (*p* < 0.05) under various activity types (Stand, Lie, Lying on the left side, Lying on the right side, Sit, Walk, Run and recovery).

**TABLE 5:** The results of different input schemes for XGBoost model in various tasks.

Inputs	Stand	Lie	Lie (Left)	Lie (Right)	Sit	Walk	Treadmill	Recovery
HR%+SBI	3.51	2.50	2.32	2.20	2.69	3.02	6.20	4.39
RD + SBI	2.69	2.27	1.76	1.95	3.40	1.89	6.02	4.52
MADs + SBI	1.17	1.43	1.24	1.22	1.36	3.41	3.64	3.61
HR%+RD + SBI	2.51	1.91	1.57	1.51	2.23	1.85	5.23	3.32
HR%+MADs + SBI	1.48	1.46	1.37	1.33	1.44	2.19	3.25	2.41
RD + MADs + SBI	1.04	1.44	1.09	1.17	1.38	1.81	3.24	2.71
HR%+RD + MADs + SBI	1.16	1.35	1.08	1.06	1.20	1.81	2.62	2.08

The results in Table 5 show that MADs is an important feature of VO_2_ estimation because MADs + SDI performed better than HR%+SDI and RD + SDI. MADs can significantly improve the accuracy of VO_2_ estimation under various activity types, because HR%+MADs + SDI and RD + MADs + SDI perform better than (*p* < 0.05) HR%+SDI and RD + SDI, as shown in Table 5.

RD is more closely related to VO_2_ than HR in rest and low-intensity exercise, because the MAE of RD + SDI is lower than that of HR%+SDI in most rest scenarios and warm-up walking. When we combined HR%, RD and SDI as the inputs for VO_2_ estimation, the results were better than (*p* < 0.05) the combination of HR% + SDI or RD + SDI. Therefore, respiratory features are beneficial to VO_2_ estimation.

Compared with a single parameter (HR%, RD, or MADs) as input, the MAE of combining multiple parameters as input to predict VO_2_ was smaller, and the stability and accuracy of the estimation results were better (*p* < 0.05), as shown in Table 5. The MAE of HR%+RD + MADs + SDI is only 1.83 ± 0.59 ml/kg/min. Unexpectedly, in the standing state, because the subjects are not familiar with the experimental process, HR% will have a negative impact on the predicted results, making the subjects nervous, and leading to changes in HR.

### Gender differences affect the accuracy of VO_2_ estimation results

In order to explore the influence of gender on VO_2_ estimation, we divided the subjects into two groups according to gender, and conducted a crossover experiment. In this section, the XGBoost regression model is still used, and the input scheme is HR% + RD + MADs + SDI.

Firstly, the LOSO cross-validation was used in the male group (Male-Male) and the female group (Female-Female) respectively. As shown in Table 6, the difference between the results of the two cross-validation is small, which may be caused by the difference in the number of subjects. Secondly, we also used Males’ (Females’) data as the training set for the XGBoost model, and the Females’ (Males’) data were as test set, which was named Male-Female (Female-Male) cross-validation test. The MAE of Male-Female and Female-Male tests during walking were similar (*p* > 0.05), as the baseline of VO_2_ value between male and female was not much different. However, the MAE during resting, the Bruce treadmill test, and recovery were bigger (*p* < 0.05) because of great difference in the muscle ratio and vital capacity between males and females.

**TABLE 6:** The effect of gender on predicted results under various tasks.

Train-test	Stand	Lie	Lie (Left)	Lie (Right)	Sit	Walk	Treadmill	Recovery
Male-Male	1.29	1.44	1.12	1.04	1.21	2.15	3.26	3.02
Female-Female	1.23	1.46	1.05	1.17	1.17	1.41	3.20	1.85
Female-Male	1.17	1.87	1.22	1.26	1.04	1.78	4.62	2.60
Male-Female	1.24	1.31	1.32	1.51	1.37	1.83	3.76	1.87

### Explore the VO_2_ estimation results at different levels of bruce treadmill test

In the Bruce treadmill test, when the input parameters were RD + MADs + SDI or HR%+RD + MADs + SDI, there was little difference in the MAE, as mentioned in Table 7. When HR% was added as an input parameter, it did not contribute to the accuracy of the VO_2_ estimation results. To further explore the effect of each level of Bruce treadmill test on the MAE, and to find out whether HR% has effect on the results, we have analyzed each stage of the test. The specific calculation results are shown in Table 7, which shows that compared with RD + MADs + SDI as input, when HR%+RD + MADs + SDI as input, the MAE at Level 1 to Level 5 decreased by 0.25 ml/kg/min, 0.11 ml/kg/min, 0.38 ml/kg/min, 0.98 ml/kg/min, and 3.69 ml/kg/min, respectively.

**TABLE 7:** Comparing the predicted result of different inputs in each level during Bruce treadmill test.

Input	RD + MADs + SBI	HR%+RD + MADs + SBI	*p* value
Level1	2.78	2.52	<0.05
Level2	3.26	3.15	<0.05
Level3	3.61	3.24	<0.05
Level4	4.47	3.49	<0.05
Level5	5.38	1.69	<0.05

The results in Table 7 shows that the MAE have no significant difference between HR%+RD + MADs + SDI or RD + MADs + SDI as model inputs during low and moderate intensity exercise (Levels 1, Levels 2, and Levels 3) (*p* < 0.05). However, during the high-intensity exercise (Levels 4 and Levels 5), there was a significant difference in MAE when RD + MADS + SDI and HR%+RD + MADS + SDI were as the input (*p* < 0.05). The feature of HR% plays an important role in predicting the accuracy of performance. Combining with Table 4, it indicates that if we intended to achieve the real-time, accurate estimation of VO_2_ in a variety of tasks, a combination of HR%, RD, MADs, and SDI was necessary.

### Advantages of the multi-parameter fusion XGBoost model

**FIGURE 3:**
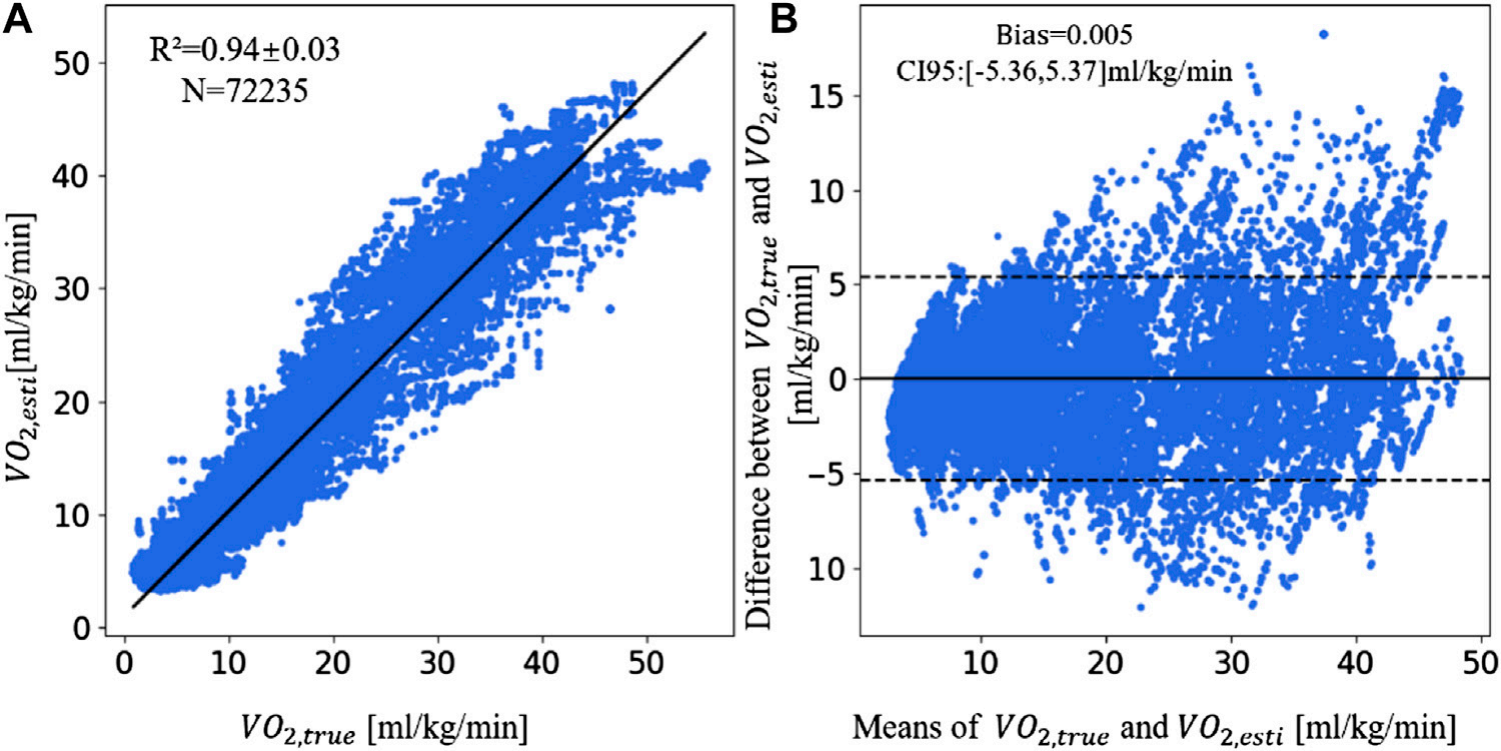
The linear correlation plot and consistency plot of $VO_{2,esti}$ and $VO_{2,true}$ . **(A)** The scatter plot of $VO_{2,esti}$ and $VO_{2,true}$ . **(B)** The Bland-Altman plot of $VO_{2,esti}$ and $VO_{2,true}$ . (Abbreviations: $VO_{2,esti}$ : the estimated VO_2_ by XGBoost model, $VO_{2,true}$ : true VO_2_).

Since the XGBoost model with feature inputs of HR% + RD + MADs + SDI performs best on the dataset, we choose this model to measure the consistency between the $VO_{2,esti}$ from SensEcho and the $VO_{2,true}$ value from Metalyzer 3B. The scatter plot and Bland-Altman plot of VO_2_ value are shown in Figure 3. Figure 3A is the scatter plot of $VO_{2,esti}$ by the wearable device and $VO_{2,true}$ by the Metalyzer 3B. The $VO_{2,esti}$ and $VO_{2,true}$ value are in a strong correlation (R^2^ = 0.94 ± 0.03, *n* = 72,235). Compared with the study of Shandhi et al., the R^2^ of this study was increased by 0.15. Figure 3B is the Bland-Altman plot. The bias (0.005 ml/kg/min) is higher than the equality line, while the CI_95_ is 5.36 ml/kg/min around the bias. More specifically, the MAE was 1.13 ml/kg/min in the rest, 2.47 ml/kg/min in walk phase, 3.09 ml/kg/min in the treadmill phase, and 2.04 ml/kg/min in the recovery phase.

It was found that MAE was lower in the resting state than the movement state. This because the baseline VO_2_ values are lower in the rest period than in the movement period. The transition process between states is not considered in the experiment. Altini et al. (2015) mentioned that transition states have an impact on the estimation results. While in our experiment, the model does not need to distinguish the type of states and it shows good estimation results throughout the experiment.

### Comparison of the proposed model with activity-specific VO2 model

In the previous paper, Altini et al. (2015) proposed activity-specific linear functions to model steady-state activities and transition-specific non-linear functions to model non-steady-state activities and transitions. The result showed that the MAE between the predicted and true results of activity-specific models based on distinguishing activity states is lower than other linear or nonlinear models.

**FIGURE 4:**
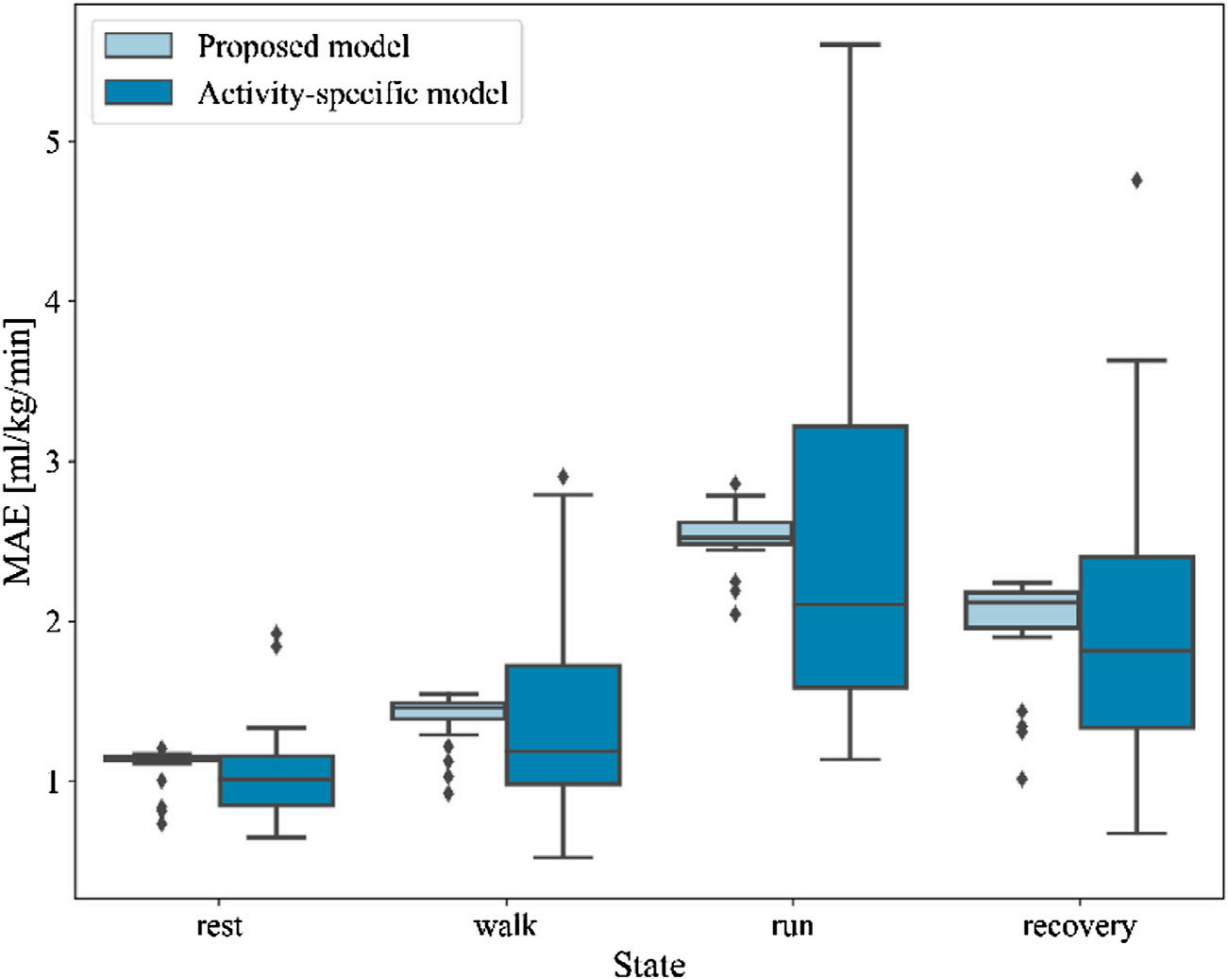
The boxplot of MAE of the proposed model and activity-specific model in different states.

In this section, we investigate the predicted results of our proposed model and the activity-specific model in the four states of rest, walk, run, and recovery, without considering the transition between states. The boxplot of MAE of two models in different states was shown in Figure 4. Compared with our proposed model, the mean MAE of the activity-specific model is close in rest (1.05 ± 0.29 vs. 1.10 ± 0.11, *p* = 0.04), walk (1.41 ± 0.15 vs. 1.40 ± 0.66, *p* = 0.69), run (2.46 ± 1.13 vs. 2.53 ± 0.17, *p* = 0.42), and recovery states (2.00 ± 0.96 vs. 2.00 ± 0.31, *p* = 0.82). The differences between two models of rest state was significant (*p* < 0.05), but that of walk, run, and recovery states were not significant (*p* > 0.05). However, the standard deviation of the activity-specific model was greater in the four states, and the model stability was slightly inferior compared with our proposed model.

### Evaluation of individual difference on estimation result

**FIGURE 5:**
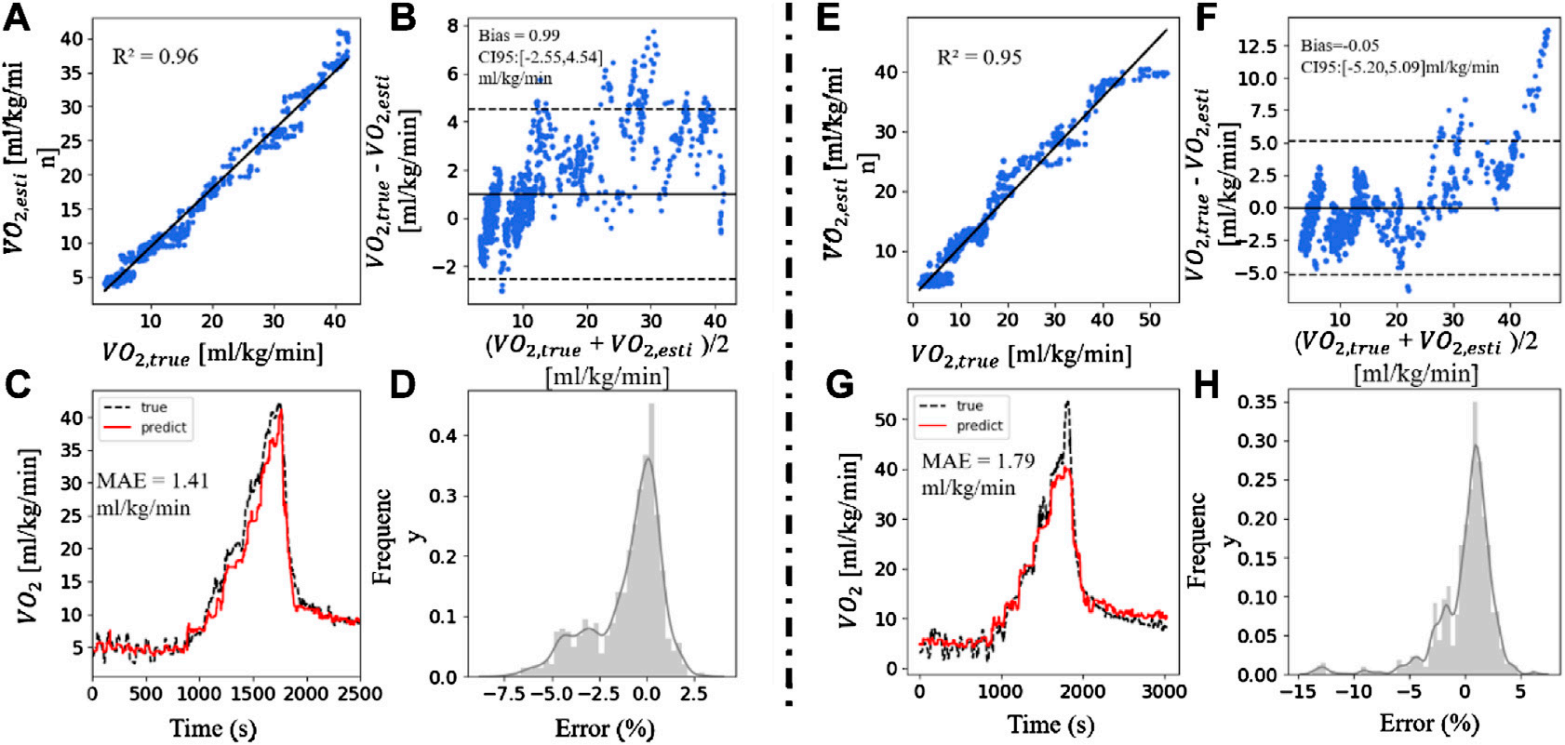
A good and a bad case. **(A,E)** The scatter plot of $VO_{2,esti}$ and $VO_{2,true}$ . **(B,F)** The Bland-Altman plot of $VO_{2,esti}$ and $VO_{2,true}$ . **(C,G)** Real-time $VO_{2,esti}$ and $VO_{2,true}$ . **(D,H)** The error distribution of $VO_{2,true}$ and $VO_{2,esti}$ .

In this section, we also used the XGBoost model with feature inputs of HR% + RD + MADs + SDI. Figure 5 showed two cases of better and worse results when applying this method to predict VO_2_. Figure 5A exhibited a strong linear correlation (R^2^ = 0.96) between the $VO_{2,true}$ and $VO_{2,esti}$ . Bland-Altman plots showed that the deviation in Figure 5B was 0.99 ml/kg/min, and the CI_95_ was (−2.55, 4.54) ml/kg/min. Figures 5C,D showed the prediction result of VO_2_ and the error distribution between $VO_{2,true}$ and $VO_{2,esti}$ during the whole experiment process. MAE was 1.41 ml/kg/min. Figures 5E–H was the result of a case of poor performance, in which R^2^ was 0.95, bias was -0.05, the CI_95_ was (−5.20, 5.09) ml/kg/min, MAE was 1.79 ml/kg/min. In this case, when the oxygen uptake rapidly rised to the peak and then falls back, our algorithm cannot accurately estimate the VO_2_ peak, but it can give the correct upward and downward trends. Both $VO_{2,esti}$ and $VO_{2,true}$ of the two subjects showed a good linear correlation, but the results were within the range of CI_95_, and the poorer subjects had a wider range. The differences in MAE was 0.39 ml/kg/min, and the basic information (age, gender, BMI) of the two groups of subjects were similar. The estimation of VO_2_ proves that differences in individual physical conditions will influence the accuracy of the results, which mostly occur when the individual’s maximum VO_2_ is close.

## Conclusion and discussion

Artificial intelligence method has great potential for predicting physiological parameters in sports medicine. Parak et al. (2017) use physiological model based on HR, running speed, and personal characteristics to estimate EE during the maximal voluntary exercise test and VO_2_max during the submaximal outdoor running test. Zignoli et al. (2022) proposed an artificial neural network that might be used to detect ventilatory thresholds for VT1 and VT2, respectively. In this work, we propose a model based on XGBoost, which uses cardio-pulmonary physiological signals as input to estimate instantaneous VO_2_ in different activity scenarios. Firstly, we explored and extracted important features from ECG, respiration, and tri-axial acceleration signals. HR%, MADs, RD and subject demography information are used as input of the XGBoost model for VO_2_ estimation. This method does not need to determine the type of exercise in advance. Compared with the linear regression method, our proposed model reduces the MAE of VO_2_ prediction by 28.79%. Secondly, the regression model trained with HR%, RD, and MADs is better than the models trained by HR%, ACC, RD, alone or in pairs. The MAE and R^2^ of LOSO cross-validation are 1.83 ml/kg/min and 0.94 respectively. Compared with the linear regression method of using single HR as input for estimation, the MAE is reduced by 54.70%. The input of the model proposed in this paper not only includes HR and RR, but also introduces MADs as input parameters, which make an important contribution to reducing the error of model prediction results. As shown in Figure 2B, MADs extracted from acceleration sensors placed on the chest can distinguish the exercise intensity especially in the Bruce treadmill test. At the same time, since the SensEcho can measure both thoracic and abdominal motion signals, we extracted RR and ${\text{DA}}_{\text{pt}}$ from respiratory signals, which can represent the changes in lung ventilation. Studies have shown that ventilation efficiency is closely related to VO_2_, and this parameter appears to be critical for patients with chronic heart failure (Figueiredo et al., 2013) and chronic obstructive pulmonary disease patients (Sanseverino et al., 2018; Fischer et al., 2021), therefore, it has great application potential. RR and ${\text{DA}}_{\text{pt}}$ are more closely related to VO_2_ than HR during resting and low-intensity exercise, which improves the prediction accuracy of our model. Interestingly, gender has little influence on results during rest and low-intensity state, as shown in Table 6, because there is little difference in the baseline of VO_2_ values between male and female. However, in the Bruce treadmill test, MAE increased significantly when the gender in the test set is different from in the training set. This reflects the difference in cardiopulmonary functions such as muscle ratio and vitality between men and women during exercise, especially during high-intensity exercise. Finally, we found that our proposed model was able to predict VO_2_ robustly with a smaller fluctuating range of error compared to the activity-specific model, the excellent model preference was attributed to the extraction of important features of the cardiorespiratory signal and the choice of model parameters.

There are limitations in our research as well. First, the number of subjects in the experiment was relatively small, the age distribution was similar, and the difference in BMI was small. The research could not investigate the influence of age, obesity, and other factors on VO_2_ estimation. In future studies, we will recruit more subjects of different ages and obesity levels to expand the sample size. Second, the model and analysis are all based on ordinary healthy subjects. There are no subjects with outstanding cardiopulmonary function such as athletes, and poor cardiopulmonary function such as patients with chronic obstructive pulmonary disease or heart failure etc. Therefore, the performance of our model on these people is unclear and needs to be further explored. Third, the type of activity is a little simple in our dataset. In the future research, we will design some combine tasks that represent the real-world situation.

In general, this study has proved the potential of cardio-pulmonary physiological signals for instantaneous estimation of individual oxygen uptake in various scenarios of activities. Furthermore, the model proposed in this paper shows high consistency with the gold standard method. The algorithm can be embedded in portable wearable devices, helping to more accurately estimate oxygen uptake in sports, clinical, and home environments. Through continuous monitoring and evaluation of oxygen uptake, it is possible to gain a deeper understanding of the individual’s cardiorespiratory health, help to make personalized health management recommendations, and improve the understanding of exercise rehabilitation and clinical treatment effect evaluation.

## Data Availability

The original contributions presented in the study are included in the article/supplementary material, further inquiries can be directed to the corresponding authors.

## References

[B1] Altini M. Penders J. Amft O. (2015). Estimating oxygen uptake during nonsteady-state activities and transitions using wearable sensors. IEEE J. Biomed. Health Inf. 20 (2), 469–475. 10.1109/JBHI.2015.2390493 25594986

[B2] Beltrame T. Amelard R. Wong A. Hughson R. L. (2017). Prediction of oxygen uptake dynamics by machine learning analysis of wearable sensors during activities of daily living[J]. Sci. Rep. 7 (1), 1–8. 10.1038/srep45738 28378815 PMC5381118

[B3] Billat L. V. (2001). Interval training for performance: A scientific and empirical practice. Special recommendations for middle- and long-distance running. Part I: Aerobic interval training. Sports Med. 31 (1), 13–31. 10.2165/00007256-200131010-00002 11219499

[B4] Bruce R. A. Kusumi F. Hosmer D. (1973). Maximal oxygen intake and nomographic assessment of functional aerobic impairment in cardiovascular disease. Am. Heart J. 85 (4), 546–562. 10.1016/0002-8703(73)90502-4 4632004

[B5] Chen T. He T. Benesty M. Khotilovich V. Tang Y. Cho H. (2015). Xgboost: Extreme gradient boosting. R package version 0.4-2 1 (4), 1–4.

[B6] Cook A. J. Ng B. Gargiulo G. D. Hindmarsh D. Pitney M. Lehmann T. (2018). Instantaneous VO2 from a wearable device. Med. Eng. Phys. 52, 41–48. 10.1016/j.medengphy.2017.12.008 29373233

[B7] CORTEX Biophysik (2021). Operator’s manual MetaLyzer® 3B[EB/OL]. https://www.procarebv.nl/wp-content/uploads/2016/11/Cortex-Metalyzer-3B_-Handleiding.pdf.

[B8] Crouter S. E. Kuffel E. Haas J. D. Frongillo E. A. Bassett D. R. Jr (2010). Refined two-regression model for the ActiGraph accelerometer. Med. Sci. Sports Exerc. 42 (5), 1029–1037. 10.1249/MSS.0b013e3181c37458 20400882 PMC2891855

[B9] Díaz V. Benito P. J. Peinado A. B. Alvarez M. Martin C. Salvo V. D. (2008). Validation of a new portable metabolic system during an incremental running test. J. Sports Sci. Med. 7 (4), 532–536. 24149962 PMC3761920

[B10] Ellis K. Kerr J. Godbole S. Lanckriet G. Wing D. Marshall S. (2014). A random forest classifier for the prediction of energy expenditure and type of physical activity from wrist and hip accelerometers. Physiol. Meas. 35 (11), 2191–2203. 10.1088/0967-3334/35/11/2191 25340969 PMC4374571

[B11] Falter M. Budts W. Goetschalckx K. Cornelissen V. Buys R. (2019). Accuracy of Apple watch measurements for heart rate and energy expenditure in patients with cardiovascular disease: Cross-sectional study. JMIR Mhealth Uhealth 7 (3), e11889. 10.2196/11889 30888332 PMC6444219

[B12] Fernandes R. Cardoso C. Silva J. Vilar S. Colcao P. Barbosa T. (2006). Assessment of time limit at lowest speed corresponding to maximal oxygen consumption in the four competitive swimming strokes[J]. Rev. Port. Ciências do Desporto 6, 128–130.

[B13] Figueiredo P. Ribeiro P. Bona R. L. Peyre-Tartaruga L. A. Ribeiro J. P. (2013). Ventilatory determinants of self-selected walking speed in chronic heart failure. Med. Sci. Sports Exerc. 45 (3), 415–419. 10.1249/MSS.0b013e318277968f 23059867

[B14] Fischer G. De Queiroz F. B. Berton D. C. Schons P. Oliveira H. B. Coertjens M. (2021). Factors influencing self-selected walking speed in fibrotic interstitial lung disease. Sci. Rep. 11 (1), 12459–9. 10.1038/s41598-021-91734-x 34127700 PMC8203722

[B15] Gastinger S. Donnelly A. Dumond R. Prioux J. (2014). A review of the evidence for the use of ventilation as a surrogate measure of energy expenditure. JPEN. J. Parenter. Enter. Nutr. 38 (8), 926–938. 10.1177/0148607114530432 24743390

[B16] Gastinger S. Sorel A. Nicolas G. Gratas-Delamarche A. Prioux J. (2010). A comparison between ventilation and heart rate as indicator of oxygen uptake during different intensities of exercise. J. Sports Sci. Med. 9 (1), 110–118. 24149394 PMC3737974

[B17] Guidetti L. Meucci M. Bolletta F. Emerenziani G. P. Gallotta M. C. Baldari C. (2018). Validity, reliability and minimum detectable change of COSMED K5 portable gas exchange system in breath-by-breath mode. PloS one 13 (12), e0209925. 10.1371/journal.pone.0209925 30596748 PMC6312326

[B18] Hamilton P. (2002). “Open source ECG analysis[C],” in Computers in cardiology, 101–104. 10.1109/CIC.2002.1166717

[B19] Hamlin M. Draper N. Blackwell G. Shearman J. P. Kimber N. E. (2012). Determination of maximal oxygen uptake using the bruce or a novel athlete-led protocol in a mixed population. J. Hum. Kinet. 31 (1), 97–104. 10.2478/v10078-012-0010-z 23486694 PMC3588657

[B20] Hill A. Lupton H. (1923). Muscular exercise, lactic acid, and the supply and utilization of oxygen. QJM Int. J. Med. 97 (62), 135–171. 10.1093/qjmed/os-16.62.135

[B21] Hills A. P. Mokhtar N. Byrne N. M. (2014). Assessment of physical activity and energy expenditure: An overview of objective measures. Front. Nutr. 1, 5. 10.3389/fnut.2014.00005 25988109 PMC4428382

[B22] Hupin D. Roche F. Gremeaux V. Chatard J. C. Oriol M. Gaspoz J. M. (2015). Even a low-dose of moderate-to-vigorous physical activity reduces mortality by 22% in adults aged ≥60 years: A systematic review and meta-analysis. Br. J. Sports Med. 49 (19), 1262–1267. 10.1136/bjsports-2014-094306 26238869

[B23] Kaminsky L. A. Arena R. Ellingsen Ø. Harber M. P. Myers J. Ozemek C. (2019). Cardiorespiratory fitness and cardiovascular disease - the past, present, and future. Prog. Cardiovasc. Dis. 62 (2), 86–93. 10.1016/j.pcad.2019.01.002 30639135

[B24] Kenny G. P. Notley S. R. Gagnon D. (2017). Direct calorimetry: A brief historical review of its use in the study of human metabolism and thermoregulation. Eur. J. Appl. Physiol. 117 (9), 1765–1785. 10.1007/s00421-017-3670-5 28689303

[B25] Lanferdini F. J. Silva E. S. Machado E. Fischer G. Peyre-Tartaruga L. A. (2020). Physiological predictors of maximal incremental running performance. Front. Physiol. 11, 979. 10.3389/fphys.2020.00979 32848890 PMC7419685

[B26] Leonard W. R. (2012). Laboratory and field methods for measuring human energy expenditure. Am. J. Hum. Biol. 24 (3), 372–384. 10.1002/ajhb.22260 22419374

[B27] Livingstone M. (1997). Heart-rate monitoring: The answer for assessing energy expenditure and physical activity in population studies?[J]. Br. J. Nutr. 78 (6), 869–871. 10.1079/bjn19970205 9497439

[B28] Lu K. Yang L. Abtahi F. Lindecrantz K. Rödby K. Seoane F. (2019). “Wearable cardiorespiratory monitoring system for unobtrusive free-living energy expenditure tracking[C],” in World congress on medical physics and biomedical engineering (Singapore: Springer), 433–437.

[B29] Makowski D. Pham T. Lau Z. J. Brammer J. C. Lespinasse F. Pham H. (2020). NeuroKit2: A Python toolbox for neurophysiological signal processing. Behav. Res. Methods 53 (4), 1689–1696. 10.3758/s13428-020-01516-y 33528817

[B30] Mazzoleni M. J. Battaglini C. L. Martin K. J. Coffman E. M. Ekaidat J. A. Wood W. A. (2018). A dynamical systems approach for the submaximal prediction of maximum heart rate and maximal oxygen uptake. Sports Eng. 21 (1), 31–41. 10.1007/s12283-017-0242-1

[B31] Meyer T. Georg T. Becker C. Kindermann W. (2001). Reliability of gas exchange measurements from two different spiroergometry systems. Int. J. Sports Med. 22 (08), 593–597. 10.1055/s-2001-18523 11719895

[B32] Montoye A. H. Vondrasek J. D. James B. Hancock I. (2020). Validity and reliability of the VO2 master pro for oxygen consumption and ventilation assessment. Int. J. Exerc. Sci. 13 (4), 1382–1401. 33042375 10.70252/THJT1177 PMC7523887

[B33] Morde V. (2019). XGBoost algorithm: Long may she reign.

[B34] Murakami H. Kawakami R. Nakae S. Yamada Y. Nakata Y. Ohkawara K. (2019). Accuracy of 12 wearable devices for estimating physical activity energy expenditure using a metabolic chamber and the doubly labeled water method: Validation study. JMIR Mhealth Uhealth 7 (8), e13938. 10.2196/13938 31376273 PMC6696858

[B35] Negus R. A. Rippe J. M. Freedson P. Michaels J. (1987). Heart rate, blood pressure, and oxygen consumption during orthopaedic rehabilitation exercise. J. Orthop. Sports Phys. Ther. 8 (7), 346–350. 10.2519/jospt.1987.8.7.346 18797046

[B36] Nevill A. M. Cooke C. B. (2016). The dangers of estimating V˙ O2max using linear, nonexercise prediction models[J]. Med. Sci. Sports Exerc. 49 (5), 1036–1042. 10.1249/MSS.0000000000001178 27922463

[B37] Nicolò A. Massaroni C. Passfield L. (2017). Respiratory frequency during exercise: The neglected physiological measure. Front. Physiol. 8, 922. 10.3389/fphys.2017.00922 29321742 PMC5732209

[B38] Parak J. Uuskoski M. Machek J. Korhonen I. (2017). Estimating heart rate, energy expenditure, and physical performance with a wrist photoplethysmographic device during running. JMIR Mhealth Uhealth 5 (7), e97. 10.2196/mhealth.7437 28743682 PMC5548984

[B39] Passler S. Bohrer J. Blöchinger L. Senner V. (2019). Validity of wrist-worn activity trackers for estimating VO2max and energy expenditure. Int. J. Environ. Res. Public Health 16 (17), 3037. 10.3390/ijerph16173037 PMC6747132 31443347

[B40] Pulkkinen A. Kettunen J. Martinmäki K. Saalasti S. Rusko H. (2004). On- and off-dynamics and respiration rate enhance the accuracy of heart rate based VO2 estimation. Med. Sci. Sports Exerc. 36 (5), S253. 10.1249/00005768-200405001-01208

[B41] Sanseverino M. A. Pecchiari M. Bona R. L. Berton D. C. de Queiroz F. B. Gruet M. (2018). Limiting factors in walking performance of subjects with COPD. Respir. Care 63 (3), 301–310. 10.4187/respcare.05768 29162719

[B42] Sasaki J. E. Hickey A. Mavilia M. Tedesco J. John D. Kozey Keadle S. (2015). Validation of the Fitbit wireless activity tracker for prediction of energy expenditure. J. Phys. Act. Health 12 (2), 149–154. 10.1123/jpah.2012-0495 24770438

[B43] Scott C. B. (2005). Contribution of anaerobic energy expenditure to whole body thermogenesis. Nutr. Metab. 2 (1), 14. 10.1186/1743-7075-2-14 PMC1182393 15958171

[B44] Shandhi M. M. H. Bartlett W. H. Heller J. A. Etemadi M. Young A. Plotz T. (2020). Estimation of instantaneous oxygen uptake during exercise and daily activities using a wearable cardio-electromechanical and environmental sensor. IEEE J. Biomed. Health Inf. 25 (3), 634–646. 10.1109/jbhi.2020.3009903 PMC8004550 32750964

[B45] Shieh S-C. Chou J. Kao Y. (2010). Energy expenditure and cardiorespiratory responses during training and simulated table tennis match[J]. Editor. Board 22, 186.

[B46] Spurr G. Prentice A. Murgatroyd P. Goldberg G. R. Reina J. C. Christman N. T. (1988). Energy expenditure from minute-by-minute heart-rate recording: Comparison with indirect calorimetry. Am. J. Clin. Nutr. 48 (3), 552–559. 10.1093/ajcn/48.3.552 3414570

[B47] Strath S. J. Brage S. Ekelund U. (2005). Integration of physiological and accelerometer data to improve physical activity assessment. Med. Sci. Sports Exerc. 37 (11), S563–S571. 10.1249/01.mss.0000185650.68232.3f 16294119

[B48] Tanaka H. Monahan K. D. Seals D. R. (2001). Age-predicted maximal heart rate revisited. J. Am. Coll. Cardiol. 37 (1), 153–156. 10.1016/s0735-1097(00)01054-8 11153730

[B49] Wang Z. Liang H. Wang J. Zang Y. Xu H. Lan K. (2021). Investigation on new paradigm of clinical physiological monitoring by using wearable devices[J]. Sheng Wu Yi Xue Gong Cheng Xue Za Zhi 38 (4), 753–763. 10.7507/1001-5515.202010021 34459176 PMC9927528

[B50] Wang Z. Yang Z. Lan K. Li P. Hao Y. Duan Y. (2022). “Development and validation of algorithms for sleep stage classification and sleep apnea/hypopnea event detection using a medical-grade wearable physiological monitoring system[C],” in International conference on wireless mobile communication and Healthcare (Cham: Springer), 166–185.

[B51] Xiong K-Y. He H. Ni G-X. (2013). Effect of skill level on cardiorespiratory and metabolic responses during Tai Chi training. Eur. J. Sport Sci. 13 (4), 386–391. 10.1080/17461391.2011.635706 23834544

[B52] Xu H. Li P. Yang Z. Liu X. Wang Z. Yan W. (2020). Construction and application of a medical-grade wireless monitoring system for physiological signals at general wards. J. Med. Syst. 44 (10), 182. 10.1007/s10916-020-01653-z 32885290 PMC7471584

[B53] Zignoli A. Fornasiero A. Rota P. Muollo V. Peyre-Tartaruga L. A. Low D. A. (2022). Oxynet: A collective intelligence that detects ventilatory thresholds in cardiopulmonary exercise tests. Eur. J. Sport Sci. 22 (3), 425–435. 10.1080/17461391.2020.1866081 33331795

